# Delayed Surgical Treatment of Distal Biceps Tendon Rupture – A Case Report

**DOI:** 10.5812/traumamon.7146

**Published:** 2012-10-10

**Authors:** Shigong Guo

**Affiliations:** 1 Department of Trauma and Orthopaedics Surgery, Wexham Park Hospital, Slough, UK

**Keywords:** Delayed Surgical Repair, Tendons, Rupture

## Abstract

**Abstract:**

Traumatic rupture of the distal biceps tendon is rare. Conservative treatment can result in reduced flexion and supination power with reduced function. This case report emphasizes the need for prompt surgical treatment and describes the possible complications of delayed surgical intervention.

## 1. Introduction

Traumatic rupture of the biceps tendon is rare. When rupture does occur, it usually involves the long head of the proximal insertion. Distal biceps tendon rupture only occurs in about 3% of all biceps tendon injuries (1). Tendon ruptures can occur at any age; however, most patients are middle aged, ranging from 30 to 60 years (2). The biceps brachii muscle flexes the elbow and supinates of the forearm. It comprises of a long head, whose origin is from the glenoid fossa, and a short head, which comes from the coracoid process, with a distal insertion on the radial tuberosity. The main mechanism of injury to the biceps is either eccentric contraction or resisted flexion of the elbow due to heavy lifting or a fall onto an outstretched hand (3). The patient usually hears or feels a "pop", and a deformity of the muscle contour of the upper arm develops. The distal tendon is normally easily palpable at the antecubital fossa. Failure to recognize a tendon rupture and treat it appropriately could result in muscle atrophy and loss of function (2).

## 2. Case Report

A 61 year old left hand dominant office worker presented to the emergency department with a painful left arm. This gentleman was a non-smoker, had no previous history of biceps muscle pain or tendonitis, he was fit and well and a keen recreational tennis player. Five days previously, this gentleman had been trying to use his left arm to catch a motorcycle that was toppling over. The patient instantaneously felt a ‘pop’ over his left upper arm and an episode of extreme pain which quickly subsided. The pain then steadily increased over the next five days when this patient presented himself at the emergency department.

On examination, although there was no apparent bruising, the patient had an obvious “reverse Popeye's sign” showing a prominence in the proximal arm due to the proximal displacement of the biceps muscle belly, indicating rupture of the distal biceps tendon. The patient was very tender in the antecubital fossa on palpation and a palpable defect was noticed – this defect was accentuated on active flexion. The patient was able to painfully flex and extend the elbow with a range of motion from 0-90 degrees, with a Medical Research Council (MRC) power score of 4/5. Active pronation was 30 degrees and active supination was 10 degrees with a MRC power score of 3/5. The patient had full sensation in all dermatomes in his arm and was vascularly intact. A broad arm sling was applied and the patient was given the next available orthopedic trauma clinic appointment which was 4 days later. He was reviewed in the orthopedic clinic in four days’ time and was advised conservative treatment with analgesia and mobilization as pain allows.

After discussing with his friends and relatives, the patient returned to orthopaedic clinic one week later enquiring about the possibility of operative treatment. The patient was subsequently referred to an upper limb surgeon for a specialist opinion. Unfortunately the patient had already booked and paid for a holiday prior to injury and decided to go on vacation in the meantime, and it was not until a further two weeks that the patient was seen again in clinic. By this time, four weeks after the initial injury, it was decided to attempt operative fixation using a suture anchor as described by Galatz (4).

In the operative room, the patient was positioned supine on an arm board. The patient was adequately prepped and draped and a tourniquet was inflated to 250 mm Hg. A single S-shaped incision was made over the antecubital fossa and careful superficial and deep dissection down to the biceps tendon was made. The distal part of the biceps tendon was found to be completely ruptured and there was no remnant of the tendon on the radial tuberosity. The tendon was short and frayed and there was difficulty in moving the biceps muscle distally. Despite releasing the adhesions around the tendon and applying a suture to the tendon stump, the stump remained 2 cm away from the radial tuberosity even with the elbow flexed to 90 degrees. The original operative plan was abandoned and the stump of the biceps tendon was sutured onto the brachialis using a polydioxanone (PDS) suture and with the elbow in 40 degrees of flexion.

Layer closure was applied and the patient was put in a backslab for three weeks. He was advised passive range of movement whilst avoiding the last 30 degrees of extension for the next three weeks and then to commence full range of movement with the aid of physiotherapy. At follow up three months after the surgery, although the wound had healed nicely and the patient was neurovascularly intact, the patient still had an obvious “reverse Popeye” deformity. The patient was able to fully flex and extend the elbow from 0 to 140 degrees actively with MRC power 5/5. However supination remained at 10 degrees with reduced power of 3-4/5. The patient had returned to his office work and could perform his activities of daily living, but he could not return to playing recreational tennis due to limited supination. After discussing with the patient in detail, because he had recovered fully flexion and extension, returned back to work full time and was able to resume his activities of daily living, it was decided not to undertake any further surgical interventions in the form of tendon grafts.

## 3. Discussion

Prompt surgical repair (ideally within 3 weeks) of a ruptured biceps tendon is usually the preferred treatment (5). Although conservative treatment can be used in the initial stages following injury (6), patients treated conservatively have been shown to have up to 30% reduction in flexion power and 40% reduction in supination power (2). In delayed repair as in this case, the tendon is often retracted and frayed making it difficult to bridge the gap from tendon stump to the radial tuberosity. In this event, a tenodesis can be performed whereby the surgeon can suture the tendon stump onto the brachialis thereby restoring flexion power but not supination. If supination is also to be restored, then a tendon graft needs to be performed to bridge the gap. Potential autograft sites can be from the iliotibial band, tensor fascia lata or tendoachilles. Allografts can also be used in distal biceps tendon repair (7), however they may be difficult to obtain in terms of harvesting the graft from donors and cryopreservation. They may also more expensive than autografts, and may carry a risk of disease transmission and tissue rejection; therefore they are not routinely used in our hospital.

## 4. Conclusions

This case emphasizes the importance of prompt early surgical treatment in suspected distal biceps tendon rupture.

## References

[1] Gilcreest EL (1933). Rupture of muscle and tendons, particularly subcutaneous rupture of the biceps flexor cubiti.. JAMA.

[2] Williams JS, Jr., Hang DW, Bach BR, Jr (1996). Distal biceps rupture in a snowboarder. Phys Sportsmed.

[3] Louis DS, Hankin FM, Eckenrode JF, Smith PA, Wojtys EM (1986). Distal biceps brachii tendon avulsion. A simplified method of operative repair.. Am J Sports Med.

[4] Galatz LM, Jani MM, Yamaguchi K (2002). Single anterior incision exposure for distal biceps tendon repair. Tech Shoulder Elbow Surg.

[5] Chillemi C, Marinelli M, De Cupis V (2007). Rupture of the distal biceps brachii tendon: conservative treatment versus anatomic reinsertion--clinical and radiological evaluation after 2 years. Arch Orthop Trauma Surg.

[6] Hetsroni I, Pilz-Burstein R, Nyska M, Back Z, Barchilon V, Mann G (2008). Avulsion of the distal biceps brachii tendon in middle-aged population: is surgical repair advisable? A comparative study of 22 patients treated with either nonoperative management or early anatomical repair. Injury.

[7] Sanchez-Sotelo J, Morrey BF, Adams RA, O'Driscoll SW (2002). Reconstruction of chronic ruptures of the distal biceps tendon with use of an achilles tendon allograft.. J Bone Joint Surg Am.

